# Association of genetic variants in the* Sirt1* and *Nrf2* genes with the risk of metabolic syndrome in a Chinese Han population

**DOI:** 10.1186/s12902-022-00965-0

**Published:** 2022-04-01

**Authors:** T. T. Tao, X. H. Lin, S. J. Tang, W. W. Gui, W. F. Zhu, H. Li

**Affiliations:** Department of Endocrinology, School of Medicine, Sir Run Run Shaw Hospital, Zhejiang University, 7th Floor, Tower I, 3 East Qingchun Road, Hangzhou, 571 ZJ China

**Keywords:** Metabolic syndrome, Single nucleotide polymorphisms, *Sirt1*, *Nrf2*

## Abstract

**Background:**

Metabolic syndrome (MetS) is a complex of interrelated risk factors, including central adiposity, increased blood pressure, hyperglycemia, elevated triglyceride levels and low high-density lipoprotein. Few studies have reported the genetic variants in the *Sirt1* and *Nrf2* genes (*Sirt1* rs7895833 A > G, *Sirt1* rs2273773 C > T and *Nrf2* rs6721961 C > A) that increase the risk of type 2 diabetes mellitus and are correlated with some glycemic and metabolic traits in the Chinese Han population.

**Methods:**

Our study recruited 141 individuals with MetS and 549 individuals without MetS to investigate the associations between three single nucleotide polymorphisms (SNPs) of *Sirt1* and *Nrf2* and the risk of MetS in a Chinese Han population using the PCR-CTPP method.

**Results:**

This research showed that the risk of MetS was 2.41 times higher for the AA genotype (*P* = 0.038) and 1.94 times higher for the AG genotype (*P* = 0.016) compared with carriers of the GG genotype. The serum levels of low-density lipoprotein cholesterol and HOMA-IR were significantly higher (*P* < 0.05) in carriers of the AA genotype of *Sirt1* rs7895833 than in carriers of the AG and GG genotypes in the general population. The serum level of total cholesterol in the AA genotype was lower (*P* = 0.033) than that in the other two genotypes. However, the genotype frequencies of *Sirt1* rs2273773 and *Nrf2* rs6721961 in the MetS group were not significantly different from those in the control subjects, and those two genetic variants were not correlated with metabolic traits.

**Conclusions:**

These results underscore the contributions of SNPs of *Sirt1* rs7895833 to MetS susceptibility as well as glycemic and metabolic traits in a Chinese population.

**Supplementary Information:**

The online version contains supplementary material available at 10.1186/s12902-022-00965-0.

## Background

Metabolic syndrome (MetS) is characterized by a cluster of traits, including central obesity, dyslipidemia, elevated blood pressure, and abnormal glucose metabolism. MetS has been found to be associated with type 2 diabetes mellitus (T2DM), cardiovascular disease and a plethora of cancers [1]. Recently, the worldwide prevalence of MetS has rapidly increased, affecting 20%-30% of the general population [2], and MetS has become a serious global public health problem. The prevalence of metabolic syndrome was recently found to be 33.9% (31.0% in men and 36.8% in women), which indicates that metabolic syndrome affects approximately 454 million adults in China [3]. Insulin resistance and central obesity are widely believed to be vital features of MetS. On the basis of family and twin studies, a genetic predisposition appears to stand out as a potential causative factor [4]. Studies on the genetic factors of MetS have currently focused on candidate genes related to lipid, glucose and energy metabolism.

Sirtuin 1 (*Sirt1*) is a longevity gene that protects cells against oxidative and genotoxic stress by deacetylating a large number of substrates, such as p53 and FOXOs [5]. Recent studies have also revealed that *Sirt1* plays an important role in glucose homeostasis and fat metabolism [6, 7]. Although previous studies have shown that some of the *Sirt1* SNPs are associated with glucose tolerance, obesity, body fat and blood pressure [8–10], there are very few studies of *Sirt1* gene SNPs and MetS in a Chinese Han population.

Furthermore, *Sirt1* is involved in the biological processes of oxidative stress and inflammation by activating the transcriptional activity of downstream genes, including nuclear-related factor 2 [11]. Nuclear factor erythroid 2-related factor 2 (*Nrf2*) is a member of the cap ‘n’ collar family of transcription factors, which play crucial roles in regulating the expression of antioxidant genes. Many SNPs in the *Nrf2* gene are predicted to affect the risk of newly diagnosed T2DM, increased blood pressure and cardiovascular mortality [12–14]. However, there is no report on *Nrf2* SNPs in Chinese MetS patients.

Our study aimed to investigate the associations between *Sirt1* rs7895833 A > G in the promoter region, *Sirt1* rs2273773 C > T in the exon 5 silent mutation and *Nrf2* rs6721961 C > A in the promoter region and metabolic syndrome risk in a Chinese Han population using polymerase chain reactions with two-pair primers (PCR-CTPP).

## Methods

### Study subjects

Our study was conducted from March to May 2010 in the Caihe community of Hangzhou, Zhejiang Province, China. The study group consisted of 690 eligible Chinese Han adults aged 40–65 years old. The Medical Ethics Committee of Sir Run Run Shaw Hospital affiliated with the School of Medicine, Zhejiang University, approved this study. Written informed consent was obtained from all participants. All subjects were familial unrelated and had no history of hyperlipidemia, hypertension or a family history of diabetes. Subjects with the following features were excluded: 1) impaired liver or renal function, 2) malignant tumors, 3) cardiovascular or peripheral vascular disease, 4) acute infectious disease or chronic inflammatory disease, 5) pregnancy, 6) thyroid disease or glucocorticoid treatment, or 7) incomplete clinical data.

### Measurements

Face-to-face interviews were conducted among the subjects by trained medical staff using a standardized questionnaire to collect information about their general sociodemographic characteristics. All participants arrived at the physical examination department of the above hospital at 7:00–8:00 am following an overnight fast. Subjects without a validated history of diabetes mellitus (DM) received a 75 g oral glucose tolerance test (OGTT), whereas a 100 g carbohydrate (steamed bread meal) test was conducted on subjects with DM [15].

Fasting plasma glucose (FPG), 2-h postprandial blood glucose (2 hPG), triglyceride (TG), total cholesterol (TC), low-density lipoprotein-cholesterol (LDL-C), high-density lipoprotein-cholesterol (HDL-C), insulin, uric acid, creatinine, urea nitrogen, urine albumin-to-creatinine ratio (UACR), uric acid (UA), serum creatinine (CREA), and serum urea nitrogen (BUN) were assayed with an autoanalyzer (Aeroset, Chicago, IL, USA). Glycosylated hemoglobin A_1_c (HbA_1_c) was measured by ion-exchange high-performance liquid chromatography (Hemoglobin Testing System; Bio-Rad, Hercules, CA, USA). Serum insulin levels were measured with a radioimmunoassay using an insulin detection kit (Beijing North Institute of Biological Technology, China). Insulin sensitivity was assessed by a homeostasis model assessment for insulin resistance (HOMA-IR) based on fasting glucose and insulin measurements as follows: [insulin (μIU/ml) × fasting blood glucose (mg/dl)/18]/22.5 [16]. Body mass index (BMI) was calculated by dividing body weight by height squared. Waist circumference (WC) was measured at the horizontal plane between the inferior costal margin and the iliac crest on the midaxillary line. Hip circumference was measured at the widest point of the hips, and the waist-to-hip ratio (WHR) was calculated and recorded for each patient. Systolic blood pressure (SBP) and diastolic blood pressure (DBP) were measured in triplicate using a mercury sphygmomanometer, and the average of the three measurements was recorded. Body fat percentage (Fat%) was measured by a bioelectrical impedance analysis system (BIA) (TBF-300, Tanita Co., Japan). MRI scans were performed at the level of the umbilicus between L4 and L5 with the subject in the supine position. Abdominal visceral fat area (VFA) and abdominal subcutaneous fat area (SFA) were calculated using SliceOmatic software (version 4.2).

### Definition of MetS

MetS was defined according to the standards generated by the Guidelines for the Prevention and Treatment of Type 2 Diabetes in China [17]. Individuals with three or more of the following abnormalities were considered as having MetS: central obesity (WC > 90 cm for men and > 85 cm for women); hypertriglyceridemia (≥ 1.70 mmol/L); low HDL-C (< 1.04 mmol/L); elevated BP (≥ 130/85 mmHg or current treatment for hypertension); and hyperglycemia (FPG ≥ 6.1 mmol/L or 2 h postprandial glucose (2 h PG) ≥ 7.8 mmol/L).

### Genotyping of *Sirt1* and *Nrf2* Gene SNPs

**Fig. 1:**
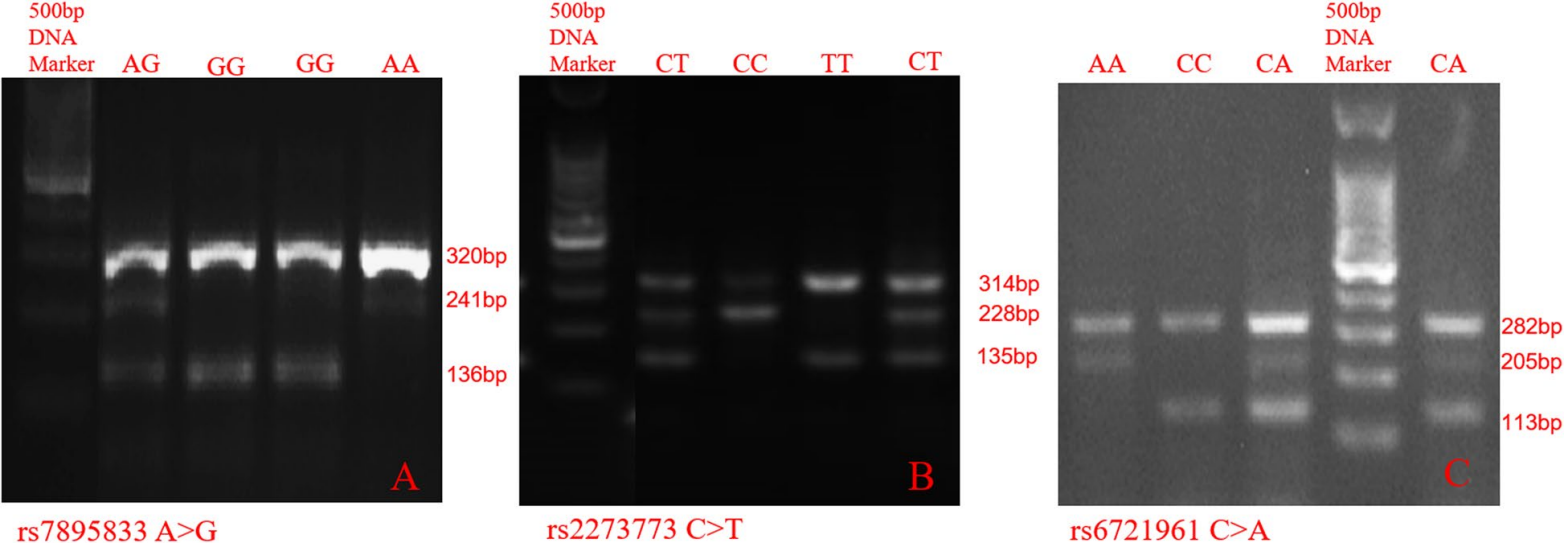
The genotype for SNPs showing in gel. **a** Representative gel showing the genotype for rs7895833 SNP of Sirt1 gene. **b** Representative gel showing the genotype for rs2273773 SNP of Sirt1 gene. **c** Representative gel showing the genotype for rs6721961 SNP of Nrf2 gene. The first lanes of each gel contain a 500 bp DNA ladder

Three tagging SNPs, namely, *Sirt1* rs2273773, *Sirt1* rs7895833 and *Nrf2* rs6721961, were selected from the HapMap database, which covered 100% of the common variations of the *Sirt1* and *Nrf2* genes in Chinese individuals. The genotyping of these SNPs was performed using polymerase chain reactions with a confronting two-pair primer (PCR-CTPP) assay [18]. Briefly, 20-μl total PCR mixtures containing 1 μl DNA, 2 μl of each primer, 10 μl of GoTaq Green Master Mix, and 7 μl of DEPC treated water in the supplied reaction buffer were prepared. The PCR was performed with the primers shown in Table 1, with the initial denaturation at 95 °C for 10 min; 35 cycles of 95 °C for 1 min., 63 °C for 1 min., and 72 °C for 1 min; and a final step at 72 °C for 5 min. The PCR products were visualized on a 2% agarose gel with Gel Red staining and then genotyped. Three genotypes for each polymorphism were defined by three distinct banding patterns. For the **rs7895833 A > G polymorphism**: 320 and 241 bp for the AA genotype; 320, 241, and 136 bp for the AG genotype; and 320 and 136 bp for the GG genotype; for the **rs2273773 C > T polymorphism**: 314 and 228 bp for the CC genotype; 314, 228, and 135 bp for the CT genotype; and 314 and 135 bp for the TT genotype; for the **rs6721961 C > A polymorphism**: 282 and 113 bp for the CC genotype; 282, 205, and 113 bp for the CA genotype; and 282 and 205 bp for the AA genotype (Fig. 1) [9, 19]. Meanwhile, a total of 2070 PCR products were sequenced by the Hangzhou Qingke Xinye Biotechnology Company, Ltd. The results of the gene sequencing were consistent with those obtained by gel imaging.

### Statistical analysis

Categorical variables are presented as frequencies and percentages. All continuous variables were tested for a normal distribution, and normally distributed variables are expressed as the mean ± standard deviation (SD). Variables with a skewed distribution are presented as the median value (interquartile range) [M(IQR)]. The Hardy–Weinberg equilibrium was tested by a goodness-of-fit χ2 test in the non-MetS group. Differences in baseline characteristics between participants with and without Mets were analyzed by t-tests for normally distributed variables and Mann–Whitney *U* tests for skewed distributed variables. Pearson’s correlation test was also applied to determine the relation between the risk of MetS and the other parameters. Binary logistic regression without or with age, sex, smoking and drinking as covariables was used to calculate the crude odds ratios (ORs) or adjusted ORs as well as the 95% confidence intervals (95% CIs) to determine the association between each SNP and the risk of MetS. Both the additive genetic model and the dominant model were estimated in the above analyses. Linear regression analysis with adjustment for age, sex, smoking and drinking was performed to test the association between each SNP and the metabolic traits. The results for the linear regression analysis are presented as the regression coefficient ± standard error (SE). All statistical analyses were performed with SPSS 22.0 (IBM, Armonk, NY, USA), and significance was defined as a *P* value of < 0.05.

## Results

### Anthropometric and metabolic characteristics of the study subjects

The anthropometric and metabolic characteristics of the 690 studied subjects at baseline are presented in Table 2. In total, 141/690 (20.4%) individuals met the criteria for MetS. As expected, participants with MetS had a greater number of adverse risk factors than participants without MetS, including a higher BMI, WHR, fat%, SFA, VFA, HOMA-IR, HbA1c, TC, UA, UACR, and MetS defining parameters (higher WC, blood glucose, TG, BP, and lower HDL-C) (*P* < 0.05 for all). However, there were no statistically significant differences in LDL-C, CREA, or BUN between the study groups (*p* > 0.05). In addition, no significant differences were observed for age, sex, smoking and drinking between the cases and controls; thus, the four variables were further adjusted in the logistic and linear regression model to control for possible confounding effects among the main effects of each SNP on the traits or disease.

### Associations between the three SNPs and the risk of MetS

The results of the association between each SNP and MetS risk are shown in Table 3. There was only a significant difference in the genotype frequency of *Sirt1* rs7895833 between the cases and controls (*P* = 0.015). Compared to the GG genotype, AG heterozygotes had an increased risk of MetS (adjusted OR = 1.937, 95% CI: 1.09–2.144,* P* = 0.016), and AA homozygotes had a further increased risk (adjusted OR = 2.414, 95% CI: 1.37–3.512, *P* = 0.038). In other words, the risk of MetS was increased by 2.41 times in the AA genotype and 1.94 times in the AG genotype compared with carriers of the GG genotype. There was no significant difference between the carriers of the A allele and the G allele (*P* = 0.675), the data is shown in Supplementary Table S1. However, for *Sirt1* rs2273773 and *Nrf2* rs6721961, no statistically significant difference was observed between the groups for the genotypes and allele frequencies (*P* > 0.05 for both).

### Associations between the three SNPs and anthropometric and metabolic characteristics

The results for the association analyses of the three SNPs with the anthropometric and metabolic characteristics are shown in Table 4. For rs7895833 of the *Sirt1* gene, there was a significantly positive correlation between the AA and AG genotypes and the level of serum LDL-C under both the additive model (regression coefficient ± standard error: 0.338 ± 0.098; *P* = 0.001) and the dominant model (0.272 ± 0.077; *P* < 0.001). The LDL-C level gradually increased from the GG genotype carriers and AG genotype carriers to the AA genotype carriers. Significant correlations were observed between the AA or AG genotype and HOMA-IR (0.023 ± 0.011; *P* = 0.035) under the dominant model, as individuals with the AA or AG genotype had visibly higher concentrations of HOMA-IR than those with the GG genotype. Meanwhile, the SNP was also significantly correlated with the TC level under both the additive model (-0.117 ± 0.055; *P* = 0.033) and the dominant model (-0.104 ± 0.043; *P* = 0.016), where individuals harboring the GG or AG genotype had a lower concentration of TC than those harboring the AA genotype. Furthermore, no other significant correlation was observed between *Sirt1* rs7895833 and the anthropometric and metabolic characteristics. For *Sirt1* rs2273773 and *Nrf2* rs6721961, there was no significant correlation between them and the anthropometric and metabolic characteristics (shown in Supplementary Table S2, S3).

## Discussion

Currently, metabolic syndrome is a global public health problem, and understanding its molecular background has gained importance worldwide [20]. There are considerable data about the relationship between the *Sirt1* and *Nrf2* genes and metabolic diseases. However, little is known about the association of genetic variants in the *Sirt1* and *Nrf2* genes with metabolic syndrome risk in a Chinese Han population. Thus, we performed our experiments on three candidate polymorphisms in the *Sirt1* and *Nrf2* genes in a Chinese Han population.

Shimoyama et al. [9] reported that A allele carriers of *Sirt1* rs7895833 had a high risk of obesity in 1,279 Japanese health checkup examinees. Zillikens et al. [21] reported that carriers of the A allele for rs7895833 had a higher BMI; therefore, carriers of this allele had an increased risk of obesity in Dutch Caucasian populations. Many researchers consider the obesity epidemic to be the main driver of the high prevalence of MetS [1, 22]. In our research, the risk of MetS was increased by 2.41 times in the AA genotype carriers and 1.94 times in the AG genotype carriers compared with carriers of the GG genotype. Based on previous studies, the A allele carriers in rs7895833 tend to be obese and therefore at high risk for MetS, which is consistent with the findings of the present study. Thus, it is conceivable to see that subjects carrying the rs7895833 AG and AA genotypes had higher levels of LDL-C and HOMA-IR than those carrying the GG genotype.

The most puzzling aspect of the findings of this study is that rs7895833 showed a negative correlation with TC. It is possible that other factors disturbed the correlation between rs7895833 and TC levels, such as lipid-lowering drugs, sample selection and a skewed distribution of data. Furthermore, the rs7895833 SNP is located in the promoter. In addition, its base sequence is TTGACT, which has been proven to be a W-box-like element of the promoter [23]. Recently, the SNP Sirt1 rs10509291, which is also located in the promoter, was proven to be closely associated with T2DM, and subjects in the Chinese Han population who were homozygous for the A allele were more likely to develop T2DM [24]. Therefore, it is likely that these polymorphisms affect the activity of the *Sirt1* gene by regulating promoter activity and thereby *Sirt1* expression. *Sirt1* deacetylates PGC-1α to promote its activity and interacts with PPAR-γ to repress its transcription [6, 7, 25, 26]. Thus, Sirt1 gene polymorphisms might affect the activities of PGC-1α and PPAR-γ and consequently might be related to risk factors for MetS. The findings of this study suggested that the rs7895833A genotype is a possible biomarker of increased MetS susceptibility.

However, for *Sirt1* rs2273773 and *Nrf2* rs6721961, there were no significant differences in the genotype and allele distributions between patients with Mets and the controls in the present study. Moreover, there was no significant correlation between the above two SNPs and the anthropometric and metabolic characteristics. Van den Berg SW et al. [27] reported that carriers of the CT genotype of *Sirt1* rs2273773 had a higher BMI than those with the TT genotype. Shimoyama et al. [19] also observed that the *Nrf2* rs6721961 polymorphism was associated with increased blood pressure in Japanese subjects. We speculated that our nonsignificant finding was primarily due to environmental factors and the limited sample size. Individual variation due to epigenetic factors can explain the variation in the observed phenotypes.

There are some limitations to the current study. The gene expression and activity levels were not determined. The studied sample size was relatively small. Based on a retrospective design, bias such as information bias and selection bias could not be ruled out. Despite these limitations, this is the first epidemiological study that associated the SNPs of the *Sirt1* and *Nrf2* genes with MetS in a Chinese Han population.

## Conclusions

In conclusion, *Sirt1* rs7895833 is associated with an increased risk of metabolic syndrome in a Chinese Han population. The *Sirt1* variant rs7895833 is associated with increased serum levels of LDL-C, HOMA-IR and TC. *Sirt1* rs7895833 might be a promising variant for screening for an increased risk of developing metabolic diseases. Our conclusion should be confirmed in Chinese patients by a replication study with a larger sample size.

**Table 1:** Primer sequences in* Sirt1* and *Nrf2 *gene polymorphisms

Polymorphisms	Primer sequences
*Sirt1* rs7895833	F1:5’-CCCAGGGTTCAACAAATCTATGTTG-3’
	F2:5’- GGTGGTAAAAGGCCTACAGGAAA-3’
	R1:5’- GCTTCCTAATCTCCATTACGTTGAC-3’
	R2:5’- CCTCCCAGTCAACGACTTTATC-3’
*Sirt1* rs2273773	F1:5’- GTGTGTCGCATCCATCTAGATAC-3’
	F2:5’- :CTCTCTGTCACAAATTCATAGCCT-3’
	R1:5’- GTAGTTTTCCTTCCTTATCTGACAG-3’
	R2:5’-CTGAAGTTTACTAACCATGACACTG-3’
*Nrf2 *rs6721961	F1:5’-CCCTGATTTGGAGGTGCAGAACC-3’
	F2:5’-GGGGAGATGTGGACAGCG-3’
	R1:5’-GCGAACACGAGCTGCCGGA-3’
	R2:5’-CTCCGTTTGCCTTTGACGAC-3’

**Table 2:** Baseline characteristics of participants according to the presence or absence of Mets

**Variables**	**MetS**	**MetS absent**	***P*** ** value**	***r***
*N* (%)	141(20.4)	549(79.6)		
Male, *n* (%)	67(47.5)	211(38.4)	0.05	-0.075
Current smoker, *n* (%)	40(28.4)	123(22.4)	0.137	-0.057
Alcohol drinker, *n* (%)	26(18.4)	115(20.9)	0.51	-0.025
Age (years)	58.01±7.76	54.32±7.57	0.357	0.157
BMI(kg/m^2^)	25.79±3.20	22.97±2.66	<0.001	0.353
WC (cm)	86.15±8.29	76.57±8.33	<0.001	0.408
WHR	0.92±0.06	0.86±0.07	<0.001	0.357
Fat% (%)	32.88±7.34	28.57±6.95	<0.001	0.226
SFA (cm^2^)	184(139.1,241.9)	152.7(113.05,199.75)	<0.001	0.18
VFA (cm^2^)	111.3(76.81,152.05)	67(45.34,102)	<0.001	0.345
SBP (mm Hg)	131.78±15.53	119.99±14.86	<0.001	0.299
DBP (mm Hg)	84.92±10.27	79.4±9.09	<0.001	0.23
FPG (mmol/L)	5.34(4.9,6.44)	4.79(4.4,5.17)	<0.001	0.327
2h PG (mmol/L)	7.98(5.45,11.06)	5.28(4.35,6.35)	<0.001	0.35
HOMA-IR	5.71(4.34,8.52)	3.57(2.75,4.77)	<0.001	0.411
HbA1_c_ (%)	6(5.5,6.4)	5.6(5.3,5.9)	<0.001	0.266
TC (mmol/L)	5.82±1.31	5.52±1.08	0.005	0.125
TG (mmol/L)	2.17(1.78,2.91)	1.22(0.9,1.55)	<0.001	0.489
HDL-C(mmol/L)	1.25±0.34	1.51±0.35	<0.001	-0.299
LDL-C(mmol/L)	2.49±0.74	2.41±0.55	0.176	0.049
UA(mmol/L)	314.98(261.49,374.41)	261.49(213.95,320.92)	<0.001	0.233
CREA(μmol/L)	70.72(61.88,79.56)	61.88(61.88,79.56)	0.211	0.048
BUN(mmol/L)	5.87±1.61	5.73±1.28	0.274	0.024
UACR(mg/mmol)	5.92(4.09,13.93)	4.47(3.03,6.83)	<0.001	0.202

**Table 3:** Associations between the SNPs of *Sirt1* and *Nrf2* gene and the risk of MetS

		**MetS ** ***N*** **(%)**	**non-MetS**^**a**^***N*(%)**	** Crude OR (95% CI)**	**Adjusted OR (95% CI)**^**b**^
rs7895833	GG	74 (52.5)	275 (51.3)	**1.00(ref.)**	**1.00(ref.)**
	AG	48 (34)	237 (41.5)	2.042(0.97-2.224)*	1.937(1.09-2.144)*
	AA	19 (13.5)	37 (7.3)	2.508(1.21-3.637)*	2.414(1.37-3.512)*
rs2273773	TT	82 (58.1)	293 (53.4)	1.00(ref.)	1.00(ref.)
	CT	51 (36.2)	231 (42.1)	0.784(0.284-1.617)	0.668(0.314-1.746)
	CC	8 (5.7)	25 (4.5)	1.120(0.486-2.582)	1.037(0.237-1.795)
rs6721961	AA	72 (51.1)	305 (54.8)	1.00(ref.)	1.00(ref.)
	CA	60 (42.5)	208 (38.4)	1.226(0.526-2.529)	1.062(0.533-1.804)
	CC	9 (6.4)	36 (6.8)	1.074(0.435-2.048)	0.955(0.459-2.012)

Bold indicated significant correlation

^a^The observed genotype frequencies of SNPs among the non-MetS were all in agreement with the Hardy–Weinberg equilibrium (*P* > 0.05 for all)

^b^Binary logistic regression with age, sex, smoking and drinking as covariables was used to calculate the adjusted ORs as well as the 95% confidence intervals (95% CIs)

^*^ *P* < 0.05

**Table 4:** Associations between the *Sirt1* rs7895833 and anthropometric and metabolic characteristics

	**AA(56)**	**AG(285)**	**GG(349)**	**Additive effect (AA VS. AG VS. GG)**	***P***_***1***_	**Dominant effect (AA+AG VS. GG)**	***P***_***2***_
BMI(kg/m^2^)	24.18±3.25	23.42±2.89	23.55±3.05	0.007±0.022	0.736	0.006±0.017	0.719
WC (cm)	80.67±8.86	78.28±9.30	78.38±9.10	0.079±0.066	0.231	-0.058±0.052	0.265
WHR	0.89±0.06	0.87±0.07	0.87±0.07	6.922±5.894	0.241	5.069±4.614	0.272
Fat% (%)	29.96±8.09	28.99±6.60	29.75±7.58	0.005±0.007	0.448	0.006±0.005	0.26
SFA (cm^2^)	157.3(116.3,224)	153.6(117.95,202.6)	161.1(119.1,211.1)	0±0.001	0.785	-0.00007±0.001	0.896
VFA (cm^2^)	81.52(61.72,112.63)	73.59(48.85,112.8)	73.4(50.53,111.25)	0±0.001	0.669	0±0.001	0.634
SBP (mm Hg)	123.12±15.79	122.07±16.01	122.55±15.51	0.001±0.003	0.568	0.00002±0.002	0.99
DBP (mm Hg)	79.45±9.34	80.81±9.97	80.47±9.33	-0.003±0.004	0.46	-0.001±0.003	0.868
FPG (mmol/L)	4.95(4.47,5.87)	4.84(4.46,5.34)	4.84(4.46,5.31)	0.039±0.037	0.298	0.011±0.029	0.702
2h PG (mmol/L)	5.36(4.35,9.4)	5.5(4.51,6.9)	5.45(4.51,7.15)	0.003±0.013	0.807	0.004±0.01	0.692
HOMA-IR	4.33(2.79,6.35)	3.96(3,5.13)	3.89(2.93,5.42)	-0.017±0.014	0.226	**0.023±0.011**	0.035*
HbA1_c_ (%)	5.65(5.33,6.08)	5.6(5.3,6)	5.7(5.4,6)	0.033±0.053	0.542	0.018±0.042	0.664
TC (mmol/L)	5.32±1.10	5.56±1.08	5.64±1.18	**-0.117±0.055**	0.033*	**-0.104±0.043**	0.016*
TG (mmol/L)	1.42(0.91,2.22)	1.31(0.96,1.89)	1.3(0.97,1.83)	-0.012±0.022	0.602	-0.01±0.017	0.563
HDL-C(mmol/L)	1.39±0.39	1.47±0.37	1.45±0.35	0.002±0.089	0.979	-0.017±0.069	0.811
LDL-C(mmol/L)	2.51±0.61	2.36±0.57	2.24±0.61	**0.338±0.098**	0.001*	**0.272±0.077**	<0.001*
UA(mmol/L)	294.2(219.9,332.8)	267.4(213.9,332.8)	273.4(219.9,329.8)	0.001±0	0.094	0±0	0.23
CREA(μmol/L)	70.72(61.88,79.56)	70.72(61.88,79.56)	61.88(61.88,79.56)	-0.004±0.002	0.088	-0.002±0.002	0.274
BUN(mmol/L)	5.87±1.32	5.64±1.36	5.84±1.35	0.022±0.02	0.256	0.025±0.015	0.111
UACR(mg/mmol)	4.73(3.33,7.75)	4.56(2.95,7.27)	4.95(3.39,8.14)	0±0	0.466	0.0001±0	0.621

Bold indicated significant correlation

^*^ *P* < 0.05

## Supplementary Information

**Additional file1: Supplementary Table S1.** Associations between the SNPs of Sirt1 and Nrf2 gene and the risk of MetS. **Supplementary Table S2.** Associations between the Sirt1 rs2273773 and anthropometric and metabolic characteristics. **Supplementary Table S3.** Associations between the Nrf2 rs6721961 and anthropometric and metabolic characteristics.

## Data Availability

The datasets generated and/or analysed during the current study are not publicly available as per the rules and regulations of Zhejiang University Medical College Affiliated Sir Run Shaw Hospital but are available from the corresponding author on reasonable request.

## Abbreviations

MetS: Metabolic syndrome
SNP: Single nucleotide polymorphism
PCR-CTPP: Polymerase chain reaction with confronting two-pair primers
T2DM: Type 2 diabetes mellitus
HOMA-IR: Homeostasis model assessment for insulin resistance
WC: Waist circumference
WHR: Waist-to-hip ratio
VFA: Visceral fat area
SFA: Subcutaneous fat area
M(IQR): Median value (interquartile range
