# Supplementary material for: Association of genetic variants in the Sirt1 and Nrf2 genes with the risk of metabolic syndrome in a Chinese Han population
Source: BMC Endocr Disord. 2022 Apr 1;22:84. doi: 10.1186/s12902-022-00965-0 (PMC8973505; doi:10.1186/s12902-022-00965-0)
Supplement: Supplementary file 1 — Additional file1: Supplementary Table S1. Associations between the SNPs of Sirt1 and Nrf2 gene and the risk of MetS. Supplementary Table S2. Associations between the Sirt1 rs2273773 and anthropometric and metabolic characteristics. Supplementary Table S3. Associations between the Nrf2 rs6721961 and anthropometric and metabolic characteristics. [file 12902_2022_965_MOESM1_ESM.docx]

Supplementary Table S1: Associations between the SNPs of Sirt1 and Nrf2 gene and the risk of MetS

|  | | MetS N(% | non-MetS N(%) | ***P*** |
| --- | --- | --- | --- | --- |
| rs7895833 | G | 196（ 69.5） | 787（ 71.7） | 0.675 |
|  | A | 86（ 30.5） | 311（ 28.3） |  |
| rs2273773 | T | 215（ 76.2） | 817（ 74.4） | 0.744 |
|  | C | 67（ 23.8） | 281（ 25.6） |  |
| rs6721961 | A | 204（ 72.3） | 818（ 74.5） | 0.712 |
|  | C | 78（ 27.7） | 280（ 25.5） |  |

**Supplementary Table S2: Associations between the Sirt1 rs2273773 and anthropometric and metabolic characteristics**

|  | **CC(33)** | **CT(282)** | **TT(375)** | **Additive effect**  **(CC VS.CT VS.TT)** | **P1** | **Dominant effect**  **(CC+CT VS.TT)** | **P2** |
| --- | --- | --- | --- | --- | --- | --- | --- |
| BMI(kg/m^2^) | 23.58±4.05 | 23.54±3.06 | 23.55±2.85 | 0.008±0.02 | 0.706 | -0.012±0.017 | 0.49 |
| WC (cm) | 77.82±11.66 | 78.49±9.37 | 78.61±8.79 | -0.081±0.062 | 0.19 | -0.066±0.053 | 0.212 |
| WHR | 0.88±0.08 | 0.87±0.07 | 0.87±0.07 | 7.925±5.515 | 0.151 | 6.174±4.678 | 0.187 |
| Fat% (%) | 29.30±9.86 | 29.12±7.0 | 29.71±7.15 | 0.005±0.007 | 0.406 | 0.008±0.006 | 0.148 |
| SFA (cm^2^) | 141.3(116.65,189.6) | 153.7(117.72,204.43) | 160.7(119.5,206.7) | 0±0.001 | 0.535 | 0±0.001 | 0.735 |
| VFA (cm^2^) | 80.42(40.68,115.80) | 73.18(52.29,112.68) | 75.31(49.88,110.9) | -0.001±0.001 | 0.13 | -0.001±0.001 | 0.238 |
| SBP (mm Hg) | 125.32±18.58 | 123.17±16.21 | 121.56±15.06 | 0.003±0.002 | 0.243 | -0.002±0.002 | 0.42 |
| DBP (mm Hg) | 81.30±8.23 | 81.12±9.77 | 80.01±9.57 | 0±0.004 | 0.963 | -0.001±0.003 | 0.719 |
| FPG (mmol/L) | 4.57(4.26,5.20) | 4.84(4.46,5.34) | 4.84(4.46,5.34) | 0.028±0.035 | 0.428 | 0.02±0.03 | 0.494 |
| 2h PG (mmol/L) | 5.23(3.99,7.51) | 5.53(4.61,7.00) | 5.45(4.51,7.15) | -0.003±0.012 | 0.806 | 0±0.011 | 0.985 |
| HOMA-IR | 3.49(2.51,5.36) | 4.00(2.96,5.29) | 3.93(2.96,5.45) | 0.007±0.013 | 0.599 | 0.007±0.011 | 0.537 |
| HbA1_c_ (%) | 5.5(5.3,5.9) | 5.6(5.4,6) | 5.6(5.3,6) | -0.022±0.05 | 0.66 | -0.035±0.042 | 0.407 |
| TC (mmol/L) | 5.55±0.74 | 5.58±1.17 | 5.59±1.14 | -0.012±0.051 | 0.813 | 0.015±0.044 | 0.731 |
| TG (mmol/L) | 1.05(0.80,2.22) | 1.25(0.96,1.82) | 1.35(0.98,1.89) | -0.007±0.021 | 0.742 | -0.011±0.018 | 0.545 |
| HDL-C(mmol/L) | 1.39±0.39 | 1.47±0.38 | 1.45±0.34 | -0.057±0.083 | 0.495 | -0.076±0.07 | 0.28 |
| LDL-C(mmol/L) | 2.40±0.37 | 2.43±0.59 | 2.43±0.62 | -0.011±0.092 | 0.905 | -0.027±0.078 | 0.733 |
| UA(mmol/L) | 273.38(231.78,395.21) | 267.44(213.95,326.87) | 273.38(219.89,332.81) | 0±0 | 0.757 | 0±0 | 0.488 |
| CREA(μmol/L) | 70.72(61.88,92.82) | 66.3(61.88,79.56) | 70.72(61.88,79.56) | -0.001±0.002 | 0.544 | -0.001±0.002 | 0.748 |
| BUN(mmol/L) | 6.83±2.08 | 5.58±1.28 | 5.81±1.29 | 0.015±0.019 | 0.433 | 0.011±0.016 | 0.471 |
| UACR(mg/mmol) | 5.31(3.10,13.41) | 4.68(3.33,7.18) | 4.72(3.09,7.74) | 0.00009±0 | 0.709 | 0±0 | 0.561 |

Supplementary Table S3: Associations between the Nrf2 rs6721961 and anthropometric and metabolic characteristics

|  | **CC(377)** | **CA(268)** | **AA(45)** | **Additive effect**  **(CC VS.CA VS.AA)** | **P1** | **Dominant effect**  **(CC+CA VS.AA)** | **P2** |
| --- | --- | --- | --- | --- | --- | --- | --- |
| BMI(kg/m^2^) | 23.14±2.46 | 23.72±3.15 | 23.47±2.95- | -0.029±0.021 | 0.169 | 0.003±0.008 | 0.753 |
| WC (cm) | 77.20±9.48 | 78.92±9.67 | 78.40±8.77 | 0.151±0.065 | 0.019 | 0.03±0.026 | 0.248 |
| WHR | 0.87±0.07 | 0.87±0.07 | 0.87±0.07 | -1.604±5.747 | 0.018 | -2.625±2.31 | 0.256 |
| Fat% (%) | 27.84±6.87 | 29.49±7.14 | 29.62±7.34 | -0.009±0.007 | 0.211 | -0.003±0.003 | 0.351 |
| SFA (cm^2^) | 163(118.7,208.85) | 152.85(118.83,202.43) | 153.7(119.9,201.85) | 0±0.001 | 0.491 | 0±0 | 0.542 |
| VFA (cm^2^) | 73.4(53.33,109.4) | 76.56(49.32,117.65) | 67.3(33.05,110.35) | 0±0.001 | 0.653 | 0±0 | 0.347 |
| SBP (mm Hg) | 119.20±13.65 | 123.50±16.0 | 122.0±15.72 | -0.000±0.002 | 0.968 | 0±0.001 | 0.657 |
| DBP (mm Hg) | 78.58±9.50 | 81.21±10 | 80.28±9.29 | 0.001±0.004 | 0.774 | 0±0.002 | 0.782 |
| FPG (mmol/L) | 4.84(4.46,5.34) | 4.90(4.47,5.34) | 4.57(4.26,5.06) | -0.094±0.036 | 0.011 | -0.028±0.015 | 0.061 |
| 2h PG (mmol/L) | 5.45(4.4,7.01) | 5.5(4.62,7.15) | 5.61(4.48,7.04) | 0.021±0.013 | 0.098 | 0.006±0.005 | 0.232 |
| HOMA-IR | 3.96(2.93,5.41) | 4.10(3.08,5.45) | 3.55(2.44,4.75) | 0.001±0.013 | 0.926 | 0.005±0.005 | 0.357 |
| HbA1_c_ (%) | 5.6(5.35,6) | 5.65(5.4,6) | 5.7(5.3,5.95) | 0.027±0.052 | 0.605 | 0.008±0.021 | 0.713 |
| TC (mmol/L) | 5.56±1.15 | 5.56±1.15 | 5.6±1.12 | -0.033±0.053 | 0.531 | 0.026±0.021 | 0.22 |
| TG (mmol/L) | 1.3(0.97,1.86) | 1.33(0.97,1.87) | 1.26(0.92,1.82) | -0.005±0.022 | 0.812 | -0.013±0.009 | 0.127 |
| HDL-C(mmol/L) | 1.39±0.28 | 1.45±0.38 | 1.47±0.36 | -0.09±0.087 | 0.3 | -0.068±0.035 | 0.051 |
| LDL-C(mmol/L) | 2.40±0.64 | 2.40±0.58 | 2.44±0.6 | -0.064±0.095 | 0.503 | -0.037±0.038 | 0.33 |
| UA(mmol/L) | 273.38(213.95,332.81) | 270.41(219.89,337.27) | 297.15(249.61,338.75) | 0±0 | 0.3 | 0±0 | 0.136 |
| CREA(μmol/L) | 70.72(61.88,79.56) | 70.72(61.88,79.56) | 70.72(61.88,79.56) | -0.004±0.002 | 0.143 | -0.001±0.001 | 0.338 |
| BUN(mmol/L) | 5.82±1.18 | 5.76±1.17 | 5.75±1.49 | 0.009±0.019 | 0.624 | 0.001±0.008 | 0.894 |
| UACR(mg/mmol) | 4.73(3.22,7.97) | 4.91(3.26,7.47) | 4.29(2.84,6.34) | 0.000±0 | 0.822 | -0.000±0 | 0.542 |
